# The United States Health Service and State Boards of Health

**Published:** 1902-02

**Authors:** 


### The United States Health Service and State Boards of Health.

In the bill providing for a National Health Service there is provision made for what has long been desired, namely, some co-operation between the national and State authorities. This would bring the two classes of service into much closer affiliation and co-operation, and would tend to strengthen the State boards of health in their relations with municipal and local boards, which at present are frequently unsatisfactory. Having authority of law the surgeon general would be enabled, when necessity arises, to call together, not necessarily the health authorities of all the States, but of such as may have a direct or pressing interest in the matter callnig for the convention. Should necessity arise for a convocation of all, the authority is provided. With regard to the collection and publication of vital statistics, it is believed that after the adoption of uniform blanks the pride of each State health organization will prompt it to see that the necessary reports are made to insure their publication in the national health bulletins published by the service.—*American Medicine.*

